# David Huntingford Malan, DM. FRCPsych

**DOI:** 10.1192/bjb.2022.99

**Published:** 2023-08

**Authors:** Jennie Malan, Maureen Kendal

Formerly Consultant Psychiatrist, Psychotherapist and Psychoanalyst, The Tavistock Clinic, London, UK


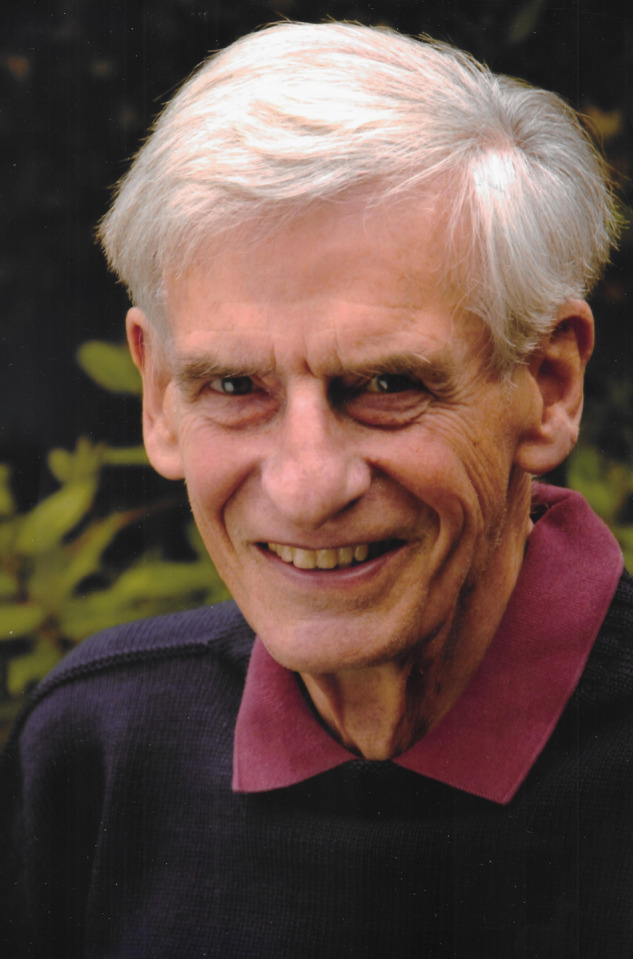


David Malan, who died on 14 October 2020 at the age of 98, worked in the Adult Department of the Tavistock Clinic between 1956 and 1982, where he led innovative research into the effectiveness of brief psychotherapy. His focus was to find a talking therapy that resolved a patient's neurosis, giving a resolution of both the current presenting and deep-seated underlying problems in a timely and cost-effective manner. He applied his research findings to therapeutic techniques and developed the ‘science of psychodynamics’, as described in his 1979 book *Individual Psychotherapy and the Science of Psychodynamics*.

David is perhaps best known for his conceptual framework of linking ‘the two triangles’. The ‘triangle of the persons’ identified the therapeutic relationship as having three significant poles: the therapist, past significant others (frequently from childhood) and present significant others. The ‘triangle of conflict’ identified attitudes of defence, hidden feelings, impulse and anxiety, neatly linking past experience, life context and the therapeutic setting.

In 1967 David set up the Brief Psychotherapy Workshop at the Tavistock Clinic. This was extremely successful and in 1992 he published the results of long-term follow-ups of 30 cases in *Psychodynamics, Training, and Outcome in Brief Psychotherapy* with Ferruccio Ossimo. He considered exploding the myth of superficiality to be his most far-reaching contribution. Critics of brief psychotherapy claimed that it could only be helpful with superficially ill patients and that only superficial improvements could be made. He maintained that ‘the aim of every session should be to put the patient in touch with as much of their true feelings as they can bear and that the long-term outcome should demonstrate deep and lasting changes’. His painstaking research repeatedly showed that brief dynamic psychotherapy could make this happen.

In 1974 Habib Davanloo visited the Tavistock Clinic to show the results of his work using intensive short-term dynamic psychotherapy. David realised that the abrasive techniques Davanloo used were not necessary; it was possible to reach the same result if a patient's defences were confronted persistently until they became exhausted, resulting in a therapeutic alliance – David adapted his approach accordingly. It was also the foundation of modern approaches to experiential dynamic therapy (EDT). They had a 12-year collaboration, summarised in Davanloo's 1995 book *Unlocking the Unconscious: Selected Papers of Habib Davanloo, MD*.

By the early 1980s, David had collaborated with colleagues at the Tavistock Clinic on a long-term follow-up study of patients who had received a variety of different types of psychodynamic talking therapy. The patients were interviewed by a team of therapists in the Adult Department of the Tavistock Clinic. The outcome data were investigated using innovative methods of computer-mediated multifactorial analysis in collaboration with Brunel University. His research into brief dynamic psychotherapy paved the way for effective psychodynamic talking therapy being offered to the public by the National Health Service (NHS) and by the International Experiential Dynamic Therapy Association (IEDTA), among others.

David was born on 21 March 1922 in Ootacamund, India, to Charles Malan, who worked in the Indian Civil Service as postmaster general of the Madras Presidency, and his American wife Isabel (née Allen). The first 7 years of his life were spent in a hill station in southern India. Then his father died from pneumonia; his vivacious mother was grief-stricken. These experiences left David with a deeply traumatic experience of personal grief and loss. He and his mother came to England, where she bought a house in Hartley Wintney which became David's beloved home for the rest of his life. He attended Winchester College with a scholarship in classics but became fascinated by science at the age of 16 and switched courses.

He won two scholarships to Oxford and graduated with a first-class honours degree in chemistry. After graduation, he was head-hunted into the Special Operations Executive, an underground army set up during the Second World War. Unfit for active service because of a foot injury, he worked on weapon development. After the war, he decided to become a psychotherapist to heal some of the war-inflicted psychological damage. He studied medicine at The London Hospital, training as a psychiatrist at the Maudsley Hospital. While at the Maudsley, he attended the Institute of Psychoanalysis, completing psychoanalytic training there.

David was a warm compassionate person who valued patients and colleagues and genuinely wanted to help them. He abhorred factionalism and enthusiastically encouraged young therapists and research assistants. He was not afraid to stand up to psychoanalytic traditions in favour of innovative approaches.

After his retirement from the NHS in 1982, David continued to research, lecture and write. He co-wrote *Lives Transformed: A Revolutionary Method of Dynamic Psychotherapy* with Patricia Coughlin, published when he was 84. This account of seven therapies demonstrated the effectiveness of intensive short-term dynamic psychotherapy in a compelling way. Always modest, David never looked for rewards or honours. He was very touched by the Career Achievement Award and tribute book presented to him at the International Congress of the IEDTA in 2005.

David balanced his busy professional life with peace and relaxation in the countryside, travelling with his wife Jennie in Scotland, New Zealand and India. He was never happier than when exploring remote, wild and beautiful places, preferably with a single-track road with no passing places along which he could drive a Land Rover!

He first married in 1959, to Muriel (née Still) and they divorced in about 1982. In 1989, he married Jennifer (Jennie) Ann (née Stead). He is survived by his wife, his son Peter from his previous marriage and three grandchildren.

